# PERCEIVED EFFICACY OF TWO NONPHARMACOLOGICAL INTERVENTIONS: A QUALITATIVE EXPLORATION OF CAREGIVERS OF DEMENTIA

**DOI:** 10.1093/geroni/igae098.2747

**Published:** 2024-12-31

**Authors:** Megumi Inoue, Catherine Tompkins, Emily Ihara, Shannon Layman, McKenzie Lauber, Sewon Kim, Gilbert Gimm

**Affiliations:** George Mason University, Fairfax, Virginia, United States; George Mason University, Fairfax, Virginia, United States; George Mason University, Fairfax, Virginia, United States; George Mason University, Fairfax, Virginia, United States; George Mason University, Fairfax, Virginia, United States; George Mason University, Fairfax, Virginia, United States; George Mason University, Fairfax, Virginia, United States

## Abstract

We will present findings from the qualitative component of an explanatory sequential design that investigated the efficacy of two nonpharmacological interventions: the Stress Busting Program (SBP) for Family Caregivers™ and a personalized music intervention for care recipients living with dementia. After implementing the 9-week SBP program focused on reducing caregiving-related stress among 97 family caregivers of persons living with dementia, we randomly assigned participants into a 4-week personalized music intervention group and a control group to examine effects on stress and well-being. Quantitative findings revealed a significant reduction in mean caregiver burden scores associated with SBP, but not for the personalized music intervention. We observed varied levels of caregiver and care recipient engagement with the personalized music intervention. To gain a deeper understanding of these results, we conducted 12 focus groups and interviews with study participants to explore caregivers’ perspectives and experiences with each intervention. Constant comparative analysis and substantive coding by two independent reviewers were employed to analyze transcripts of audio recordings with 47 caregivers, selected from the initial study cohort. The theme of bonding/connecting with group members emerged as the most effective SBP element. Also, caregivers valued the opportunity to establish a sense of community by connecting with other caregivers within SBP. However, some caregivers experienced technological difficulties with using an MP3 player for the music intervention. We will present cross-cutting themes and overall lessons learned about strategies to enhance the use of these nonpharmacological interventions.

